# Anévrisme rompu de l’artère gastroduodénale révélé par des hématémèses: à propos d’un cas

**DOI:** 10.11604/pamj.2020.37.244.22380

**Published:** 2020-11-17

**Authors:** Hicham Belmir, Youssef Tijani, Chafik El Kettani, Adil El Ghanmi, Hassan Chtata, Mustapha Taberkant

**Affiliations:** 1 Département de Chirurgie Vasculaire, Université Mohammed VI des Sciences de la Santé, Casablanca, Maroc,; 2 Département d’Anesthésie-Réanimation, Université Mohammed VI des Sciences de la Santé, Casablanca, Maroc,; 3 Département de Gynécologie et Obstétrique, Université Mohammed VI des Sciences de la Santé, Casablanca, Maroc,; 4 Département de Chirurgie Vasculaire, Université Mohammed V, Rabat, Maroc

**Keywords:** Anévrisme, artère gastroduodénale, hématémèses, Aneurysm, gastroduodenal artery, hematemesis

## Abstract

L'anévrisme de l'artère gastroduodénale est une lésion vasculaire rare, asymptomatique dans la majorité des cas. Cependant, en cas de rupture de l’anévrisme, le pronostic est mauvais et la mortalité peut atteindre 40%. Nous rapportons le cas d’un patient âgé de 83 ans, qui a présenté brutalement des douleurs abdominales non spécifiques, associées à des hématémèses, et chez qui une fibroscopie a révélé une formation battante comprimant le bulbe duodénal avec un saignement actif, une tomodensitométrie abdominale a été réalisé et a mis en évidence un anévrisme de l’artère gastroduodénale englobant son ostium, et rendant une prise en charge endovasculaire impossible. Une chirurgie ouverte a donc été réalisée, et a consisté en une mise à plat associée à une exclusion de l’anévrisme, puis complétée par une plastie du bulbe. Une tomodensitométrie post-opératoire a confirmé l’exclusion totale de l’anévrisme avec la conservation de la circulation hépatique.

## Abstract (en)

Gastroduodenal artery aneurysm is a rare vascular lesion, asymptomatic in the majority of cases. However, ruptured aneurysm is associated with poor prognosis and mortality can achieve a 40% rate. We here report the case of an 83-year-old patient with abrupt onset of non-specific abdominal pain associated with hematemesis. Endoscopy showed beating formation compressing the duodenal bulb with active bleeding. Abdominal computed tomography (CT) scan was performed which objectified gastro duodenal artery aneurysms involving the artery ostium and making endovascular treatment impossible to perform. Thus, the patient underwent open surgery based on flattening associated with aneurysm exclusion and then complemented by bulb plasty. Post-operative CT scan confirmed total exclusion of the aneurysm with preservation of hepatic circulation.

## Introduction

Les anévrismes des artères viscérales sont rares avec une incidence entre 0,1% et 2% [1-3]. Ces lésions représentent une urgence médicale dans 22% des cas, avec une mortalité de 8,5% [4]. Les anévrismes de l’artère gastroduodénale sont les moins fréquents et représentent moins de 1,5% de tous les anévrismes des artères splanchniques [5]. Ils sont souvent asymptomatiques découvert accidentellement par un échodoppler ou une tomodensitométrie abdominale, mais peuvent aussi se révéler par des symptômes mineurs sous forme de douleurs abdominales non spécifiques, soit des symptômes majeurs tel que la rupture qui constitue leur mode de révélation le plus fréquent [6] avec parfois une instabilité hémodynamique, hématémèses et mélénas. La physiopathologie des anévrismes est souvent associée à l’athérosclérose, la fibrodysplasie (Ehlers Danlos ou syndrome de Marfan), ou due à des variations hémodynamiques (hypertension portale par exemple). Les autres étiologies concernent les faux anévrismes qui peuvent être infectieuses, inflammatoire, post-traumatique [7].

Le traitement peut être chirurgical avec une mise a plat de l’anévrisme avec ou sans résection, ou ligature de l’artère avec ou sans rétablissement de continuité artérielle. La prise en charge endovasculaire comprend soit une embolisation directe de l’anévrisme par des coils, soit indirecte en embolisant toutes les afférences et efférences artérielles anévrismales, pour maintenir la perméabilité artérielle, la mise en place d’un stent couvert est possible si l’anatomie artérielle le permets [8].

Nous rapportons le cas d’un anévrisme rompu de l’artère gastro-duodénale compliqué d’une hémorragie digestive chez un patient de 50 ans, et qui a été traite avec succès par une mise à plat - ligature artérielle.

## Patient et observation

**Figure 1:**
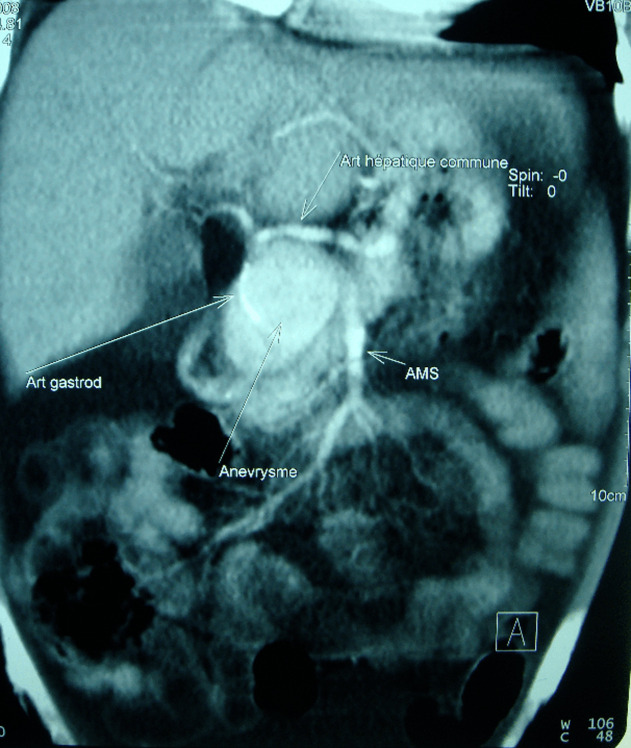
image pré-opératoire d’une reconstruction 3D d’un angioscanner abdominal montrant l’anévrisme aux dépens de l’ostium de l’artère gastro-duodénale, et au contact du bulbe duodénal

**Figure 2:**
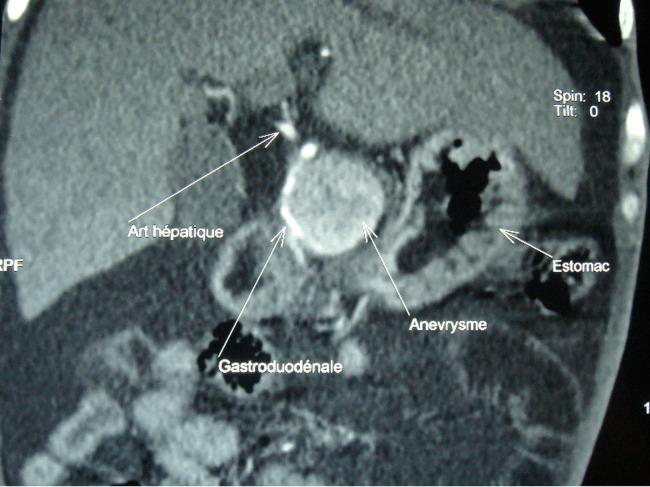
image de l’angioscanner montrant l’anévrisme de l’artère gastro-duodénale refoulant le bulbe duodénal

**Figure 3:**
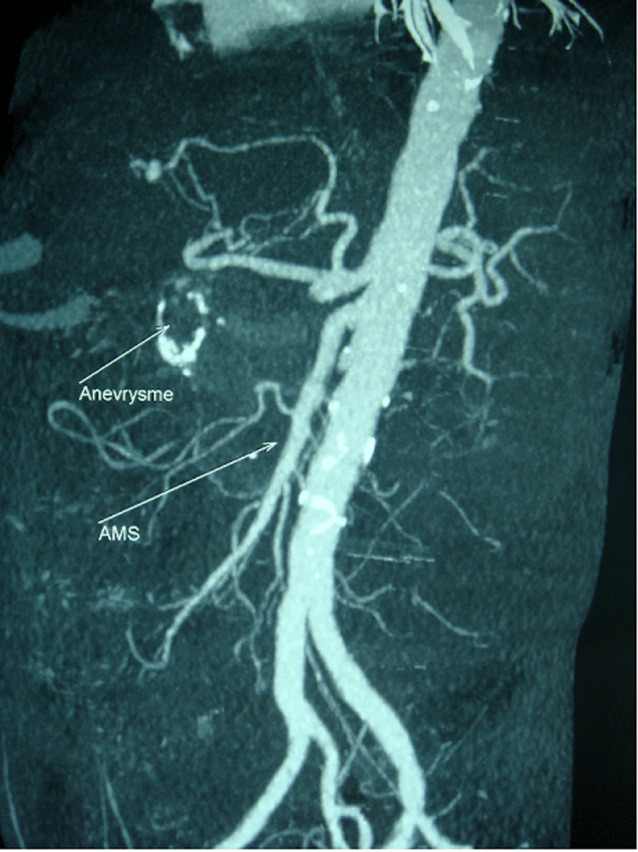
image post-opératoire d’une reconstruction 3D de l’angioscanner post-opératoire avec une vue latérale gauche montrant l’exclusion totale de l’anévrisme avec présence de calcifications au niveau de la paroi anévrismale

**Figure 4:**
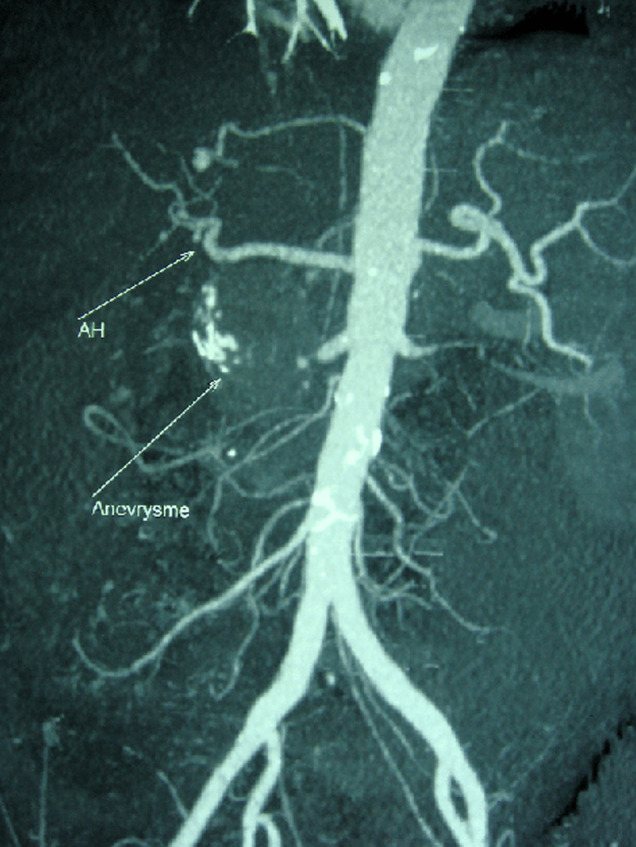
image de la même reconstruction 3D de l’angioscanner post-opératoire montrant la préservation de la circulation hépatique

Patient de 83 ans, tabagique chronique, avec des antécédents de diabète type 2 et une hypertension artérielle, admis aux urgences pour des douleurs abdominales atypiques associées à des hématémèses de grande abondance. L’examen physique a montré un patient en mauvais état général, pâle avec des conjonctives décolorées, une tachycardie et une hypotension artérielle et un abdomen souple à la palpation. Une fibroscopie œso-gastroduodénale a révélé un saignement actif provenant d’une formation battante qui refoule la face postérieure du bulbe duodénal. L’angioscanner abdominal a révélé un anévrisme de 6 cm de diamètre de l’artère gastroduodénale, englobant son ostium proximal (Figure 1) en contact étroit avec la face postérieure du duodénum (Figure 2), et rendant impossible toute possibilité de prise en charge endovasculaire. Une chirurgie ouverte a donc été réalisée, le malade a été opéré par une laparotomie médiane à cheval sur l’ombilic, l’anévrisme a été incisé longitudinalement. Un gros thrombus mural a été retiré puis les ostia de l’artère gastroduodénale proximale et distale ont été ligaturés. L’intervention a été complétée par une plastie du bulbe duodénal. Les suites opératoires étaient simples. L’étude histologique de la paroi anévrismale était en faveur de remaniements athéroscléreux, l’angioscanner post-opératoire a confirmé l’exclusion totale de l’anévrisme (Figure 3), avec la conservation de la circulation hépatique (Figure 4).

## Discussion

Bien que considérés comme rares, les anévrismes viscéraux sont parfois révélés par des complications graves, principalement représentées par les ruptures, qui ont des taux de mortalité de l'ordre de 20 à 70%, selon le site [9]. Les anévrismes de l’artère gastroduodénale s'intègrent souvent dans un contexte inflammatoire. Ils sont considérés comme une complication fréquemment attribuée à l’action enzymatique de la pancréatite aiguë ou chronique [10]. Ainsi, l'activité des médiateurs inflammatoires et des enzymes pancréatiques protéolytiques provoque la destruction des parois des vaisseaux de la région, conduisant à la formation de pseudo-anévrismes dans la majorité des cas [11]. Ils peuvent aussi être associés à une angiocholite, un traumatisme, une sténose du tronc cœliaque ou des causes iatrogènes [9]. L'abus d'alcool, une cholécystectomie antérieure, des variantes congénitales, un syndrome de Marfan, une polyartérite noueuse, une dysplasie fibromusculaire et la cirrhose hépatique ont également été décrits comme des facteurs associés [9].

Les anévrismes non rompus de l’artère gastroduodénale se manifestent par une symptomatologie vague et peu spécifique, à type de douleurs abdominales atypiques, nausées et vomissements, masse abdominale pulsatile à l’examen clinique [12,13]. La rupture des anévrismes gastroduodénaux constitue un tableau dramatique qui est malheureusement inaugurale dans 50 à 60% des cas [14]. La symptomatologie clinique, dépend essentiellement de son type. Ainsi, une hémorragie digestive extériorisée à type d’hématémèses ou de mélénas constitue un mode de découverte rare, le diagnostic est alors fait grâce à la fibroscopie œso-gastroduodénale puis la réalisation d’un angioscanner abdominal. L’artériographie peut être réalisée si le malade est stable, elle a ainsi un but à la fois diagnostique et thérapeutique [9]. La rupture intra-péritonéale constitue le mode le plus fréquent, et se manifeste par un tableau d’abdomen aigue avec une instabilité hémodynamique sévère, la rupture dans la voie biliaire est révélée par une hémobilie. La rupture rétropéritonéale se manifeste par une hypotension artérielle avec un état hémodynamique initialement stable et des douleurs abdominales vagues. Le traitement chirurgical, dans notre cas, est le traitement de choix car on est en présence d’une anatomie défavorable avec une localisation anévrismale qui intéresse l’ostium de l’artère gastroduodénale, en plus du tableau urgent de rupture entrainant un saignement actif avec une instabilité hémodynamique. La prise en charge chirurgicale consiste en une ligature de l’artère gastroduodénale après résection complète de l’anévrisme, cette technique est la plus pratiquée dans la littérature: environ 63% des cas de l’analyse anglaise publiée par Moore et ses collaborateurs [15].

Le traitement endovasculaire est préféré en cas d’anévrismes viscéraux non compliqués de rupture, avec un état hémodynamique stable, et en présence d’une anatomie favorable permettant l’embolisation, il est efficace, peu invasif avec un risque péri et post-opératoire plus faible. Le traitement chirurgical peut être complémentaire Cependant, aucun essai contrôlé randomisé n'existe pour confirmer la supériorité de la chirurgie endovasculaire ou ouverte [16].

## Conclusion

Cette observation démontre que, même s’il s’agit d’une complication rare, une hémorragie digestive peut révéler un anévrisme rompu de l’artère gastroduodénale. La chirurgie ouverte constitue le traitement de choix en cas d’instabilité hémodynamique, ou en présence d’une anatomie défavorable rendant difficile un cathétérisme vasculaire. L’embolisation est de plus en plus pratiquée dans les anévrismes viscéraux, mais elle est réservée aux cas non compliques, avec une anatomie favorable, permettant d’éviter ainsi les risques d’une dissection chirurgicale extensive, et limitant la résection des organes de voisinages à visée hémostatique.
